# Comparison of the sequences of the viral capsid protein 1 and viral capsid protein 2 encoded genes in symptomatic and asymptomatic cases of canine parvovirus 2 in dogs

**DOI:** 10.14202/vetworld.2025.8-14

**Published:** 2025-01-09

**Authors:** Mohammed Al-Saadi, Amer Nubgan, Ali Hadi Abbas

**Affiliations:** 1 Department of Internal and Preventive Medicine, College of Veterinary Medicine, University of Al-Qadisiyah, Al-Diwaniyah, Iraq; 2 Department of Biology, College of Science, University of Baghdad, 10071, Iraq; 3 Department of Microbiology, Faculty of Veterinary Medicine, University of Kufa, 54003, Najaf, Iraq

**Keywords:** canine parvovirus-2, capsid encoded genes, mutations

## Abstract

**Background and Aim:**

Canine parvovirus 2 (CPV-2) is a highly contagious virus that infects wild and domestic canines. Despite the use of a routine vaccination protocol, it is endemic in Iraq. The genetic drift of CPV-2 is a major issue worldwide because it abrogates virus control. In Iraq, there is a knowledge gap regarding the genetic sequences of asymptomatic and symptomatic CPV-2 cases. Therefore, this study aimed to perform a genetic analysis of viral capsid protein 1 (*VP1*) and viral capsid protein 2 (*VP2*), two major capsid-encoding genes, to demonstrate the possible role of certain mutations in triggering infection.

**Materials and Methods:**

Symptomatic and asymptomatic cases (n = 100/each) were tested by a polymerase chain reaction targeting *VP1* and *VP2* genes.

**Results:**

The analysis revealed numerous synonymous and nonsynonymous mutations in *VP1* and *VP2* and in the intergenic sequence.

**Conclusion:**

The study identified significant genetic mutations in *VP1, VP2*, and the intergenic regions of CPV-2 in symptomatic and asymptomatic cases in Iraq. These mutations may contribute to the virus’s ability to evade control measures such as vaccination. These findings indicate that CPV-2 polymorphisms can influence the clinical state of the disease and/or trigger infection. Understanding these genetic variations provides critical insights into CPV-2 pathogenesis and could inform improved vaccination strategies to mitigate the virus’s impact in endemic regions.

## INTRODUCTION

Parvovirus is a contagious virus that infects canines, causing severe mucoidal hemorrhagic diarrhea resulting from enteritis; it is characterized by high morbidity and mortality rates [1]. The disease is caused by canine parvovirus 2 (CPV-2). It is a non-enveloped virus that contains single-strand DNA approximately 4000–6000 bp in size. It belongs to the *Parvoviridae* family, subfamily *Protoparvovirus* carnivoran 1 species [2].

CPV-2 has spread globally with high morbidity and mortality. Iraq, in particular, is endemic to CPV-2, and infection mainly occurs due to the failure of the currently recruited vaccine as it depends on using killed or attenuated vaccines from different manufacturers. All vaccines are imported from countries like Belgium, the USA, and the Netherlands. The failure of such vaccination programs likely resulted from the genetic variability of the virus [1, 3]. Viral capsid protein 1 (*VP1*) and viral capsid protein 2 (*VP2*) are encoded by a large open reading frame through alternative splicing. *VP2* is abundantly polymorphic. Thus, numerous strains of CPV-2 have emerged since its discovery [4, 5]. These two genes are located within a major polyprotein-encoding gene in which *VP1* is 78 bp in size and is located between 2285 bp and 2362 bp, whereas the size of VP2 is 1755 bp within 2783–4537 bp. Both play pivotal roles in viral pathogenicity and immunogenicity [6]. Three antigenic variants, namely a, b, and c, are recorded globally; in Iraq, a and b variants have already been recorded [7]. However, a recent study using a complete genome sequence revealed CPV-2b and CPV-2c [8].

These variants can infect not only dogs but also wild animals [9]. It had been explained the emergence of new strains due to genetic polymorphisms of *VP2* [10]. We assumed that mutations in *VP1* and *VP2*, which are structurally encoded genes, likely exist in Iraq, leading to the failure of the control program. Thus, it is beneficial to genetically analyze these genes in acute and subclinical forms of infection to demonstrate the role of amino acid changes in the onset of infection. Importantly, genetic mutations within these genes were demonstrated, which could explain their role in the development of acute forms of infection. The clarification of these perspectives could help in disease prevention and/or the development of suitable vaccines for the disease.

## MATERIALS AND METHODS

### Ethical approval

This study was approved by Veterinary Medical Ethics Committee of the University of Al-Qadisiyah/College of Veterinary Medicine (approval number 2709 on February 07, 2024). Sampling was conducted by a trained person.

### Study period and location

The study was carried out from February 2024 to July 2024 at Al-Qadisiyah province, Iraq.

### Sample collection

Blood samples (3 mL) were collected from the saphenous veins of domestic dogs (n = 100) with typical signs of parvovirus infection. Similarly, 100 samples from stray dogs were also collected. All the procedures in this regard were carried out according to the recommended ethical aspects with all the clinical precautions of animal control and management, using specialized cages and mouth gags. The typical clinical signs of symptomatic dogs include hemorrhagic diarrhea and fever. In contrast, asymptomatic dogs were selected after noticing normal behavior and healthy clinical signs such as eating, drinking with normal feces, and body weight. The obtained blood was kept in an ethylenediaminetetraacetic acid (EDTA) tube (Afeco, Jordan) transferred to the diagnostic laboratory immediately, and stored at −20°C until further processing.

### Genomic DNA extraction

Genomic DNA was extracted from blood with anticoagulant using a commercial kit manufactured (AddBio, South Korea) according to the manufacturer’s instructions, which included lysing 200 μL of blood with 20 μL of proteinase k (20 mg/mL) and incubated at 56°C for 10 min with occasional mixing. The released DNA was purified from the generated lysate through a silica gel column. The bound DNA with the silica was washed twice with a washing buffer and finally eluted with 100 µL of high-purity polymerase chain reaction (PCR) water. The extracted DNA concentration was measured using the QuantiFluor technique (Promega, USA) and stored at −20°C until PCR.

### PCR

#### Primer design

**Figure 1:**
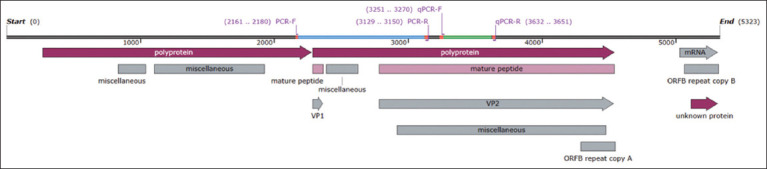
The genomic region of CPV-2 shows the location and coverage sequence of the designed primers for conventional PCR and qPCR techniques (accession number: M19296). CPV-2=Canine parvovirus 2, PCR=Polymerase chain reaction, qPCR=Quantitative polymerase chain reaction.

In this study, conventional PCR was used, and primers were designed manually (Table 1) to target the CPV-2 capsid protein, as well as the *VP1* and *VP2*-encoding genes. The primers used for conventional PCR cover the polyprotein-encoding region, including the complete region of *VP1*, the partial sequence of *VP2*, and the intergenic sequence known as phospholipase-A2 (Table 1 and Figure 1).

**Table 1:** Oligonucleotides designed for PCR and qPCR.

Primer name	Sequence ‘5→3’	Start	End	Tm	Accession number	Amplicon bp
Conventional PCR						
PCR-F	TGGCGTTACTCACAAAGACG	2161	2180	58	M19296	990
PCR-R	TTAAACCAAACTCCCCAAGCAT	3129	3150	57		

PCR=Polymerase chain reaction, qPCR=Quantitative polymerase chain reaction

#### Conventional PCR and sequencing

**Figure 2:**
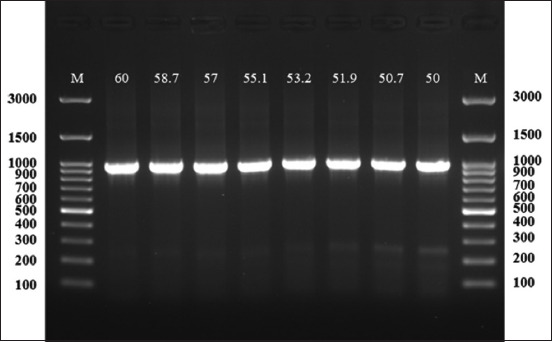
Agarose (1.5%) gel electrophoresis was performed through a gradient protocol at different annealing temperatures (60°C–50°C). This exhibited an optimal amplification in all temperatures.

Amplification of the targeted region using the designed primers (Table 1) was conducted using a thermal cycler (Bio-Rad, USA). The protocol was initially optimized using the gradient method (Figure 2). The samples were subjected to the following conditions: Initial DNA separation at 95°C for 10 min, followed by 40 cycles of 95°C for 20 s as the denaturation step, then 60°C for annealing, and extension at 72°C for 35 s. Finally, 72°C for 5 min was used as the extension. These steps were performed in a 0.2 mL PCR tube that contained 50 μL total PCR reaction, including 20 μL of Taq master mix, 2 μL of each forward and reverse primer, 3 μL of template DNA, and 23 μL of PCR H_2_O. The amplicons were confirmed by agarose gel electrophoresis (1.5 % agarose) dissolved in Tris–Borate–EDTA solution and heated. Once it reached 60°C, a safe gel stain (20 μL) (AddBio) was added, and the mixture was poured into a gel tray containing the comb. After solidification, 6 μL of each PCR product was loaded into each well and electrophoresed at 110 V and 90°C for 60 min. The gel was visualized and imaged using a gel documentation system (Syngene, Taiwan). Successful specific amplicons from symptomatic (n = 10) and asymptomatic (n = 10) dogs were sequenced bidirectionally using the Sanger technique (Macrogen, South Korea). Moreover, the obtained sequences were trimmed and analyzed using Mega X software (https://www.megasoftware.net/) by maximum likelihood (Tamura-Nei model) with 1000 bootstraps.

## RESULTS

### Conventional PCR

**Figure-3:**
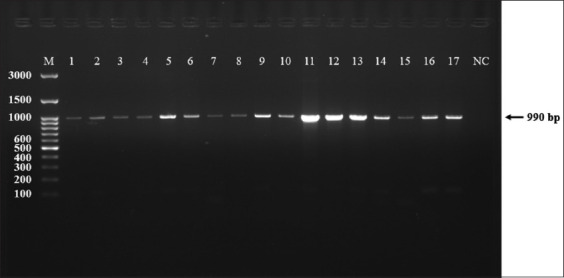
Agarose (1.5%) gel electrophoresis shows the amplified sequence of CPV-2 (990 bp); samples 1–17. NC is the negative control, whereas M is a molecular ladder (GeneDireX, South Korea). CPV-2=Canine parvovirus 2.

The gradient PCR protocol resulted in an optimal annealing temperature of 60°C. This allowed us to test all samples successfully and reveal an efficient amplicon (990 bp) (Figures 2 and 3). Among 100 samples from asymptomatic cases (Stray dogs), 91 (90%) were positive for CPV-2, whereas 100% of the symptomatic cases (domestic dogs = 100) were found positive by PCR.

### Sequence analysis

**Figure-4:**
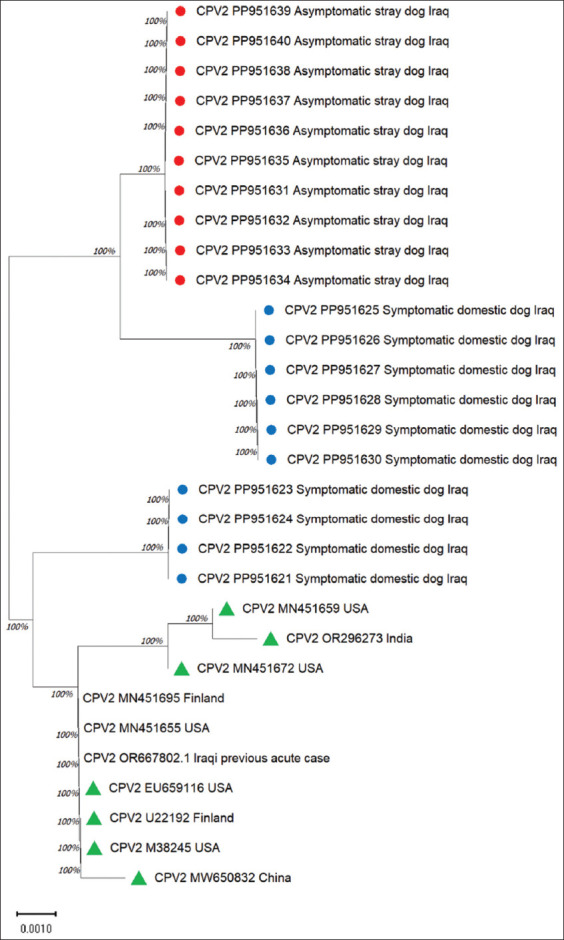
Evolutionary tree generated by the maximum likelihood method (Tamura-Nei model). This was drawn to scale, with branch lengths measured in the number of substitutions per site in 864 bp positions; the red circles represent sequences originating from asymptomatic cases, whereas the blue circles represent symptomatic cases. These strains were clustered with global strains and are referred to as green triangles.

The resulting sequences were analyzed and submitted to the National Center for Biotechnology Information. Relevant accession numbers were obtained for the symptomatic cases, including PP951621, PP951622, PP951623, PP951624, PP951625, PP951626, PP951627, PP951628, PP951629, and PP951630, while the accession numbers for the asymptomatic cases were PP951631, PP951632, PP951633, PP951634, PP951635, PP951636, PP951637, PP951638, PP951639, and PP951640. The similarity percentages were high compared with those of other global strains, ranging between 99.54% and 99.31%. The tree was grouped into 3 clusters. The cluster, including all sequences originating from asymptomatic cases of stray dogs, was separated from symptomatic cases with high confidence. However, symptomatic cases were clustered into two different clades with high support: The first clade (n = 6) with accession numbers: PP951625, PP951626, PP951627, PP951628, PP951629, and PP951630, while the rest were clustered in a clade close to the sequences isolated from different countries (Figure 4). The remaining sequences originating from the symptomatic cases were divergent from the analyzed global strains.

Notably, the identified nucleotide substitutions were synonymous and mostly nonsynonymous in the symptomatic cases (Table 2). Most mutations detected in the symptomatic cases were located in VP2; however, the translated amino acid did not mutate and was still valine. There was one mutation within *VP1*, with codon Guanine, Thiamin, and Adenine (GTA) mutated into GTG, and two substitutions within the polyprotein non-specific region between *VP1* and *VP2*, all of which appeared nonsynonymous. More importantly, five synonymous mutations were detected in the symptomatic cases that caused amino acid alterations, three of which were located within *VP2*, such as arginine (R) > lysine (K), asparagine (N) > lysine (K), and alanine (A) > glycine (G) within amino acid locations: 247, 260, and 172, respectively; furthermore, two mutations were located within the non-specific polyprotein region as cysteine (C) > tyrosine (Y) and isoleucine (I) > valine (V) at amino acid locations 31 and 34, respectively.

**Table-2:** The identified mutations within symptomatic and asymptomatic cases compared with the reference genome (accession number: M19296).

Targeted sequence	Original > mutated codon	Translated amino acids	Mutation site	Mutation type
Symptomatic cases				
VP2	TTC > TTT	Phenylalanine (F>F)	212	Nonsynonymous
VP2	AGA > AAA	Arginine (R) > Lysine (K)	247	Synonymous
VP2	GTG > GTA	Valine (V) > Valine (V)	249	Nonsynonymous
VP2	AAC > AAA	Asparagine (N) > Lysine (K)	260	Synonymous
VP1	GTA > GTG	(V) > (V)	16	Nonsynonymous
Unspecified	TGT > TAT	Cysteine (C) > Tyrosine (Y)	31	Synonymous
Unspecified	ATA > GTA	Isoleucine (I) > Valine (V)	34	Synonymous
VP2	GCA > GGA	Alanine (A) > Glycine (G)	172	Synonymous
VP2	TTC > TTA	(F) > F	212	Nonsynonymous
VP2	GAA > GAG	Glutamic acid (E) > E	227	Nonsynonymous
VP2	GTA > GTG	(V) > (V)	236	Nonsynonymous
Asymptomatic cases				
Unspecified	GCC > ACC	Alanine (A) > Threonine (T)	155	Synonymous
VP2	GCA > GGA	(A) > (G)	172	Synonymous
VP2	TTC > TTT	(F) > (F)	212	Non-synonymous
VP2	GAA > GAG	(E) > (E)	227	Non-synonymous
VP2	GTA > GTG	(V) > (V)	236	Non-synonymous

Five mutations were detected in the asymptomatic cases, but only two of them were found to be synonymous mutations as alanine (A) > threonine (T) at the nonspecific polyprotein region and alanine (A) > glycine (G) at the *VP2* located at amino acid numbers 155 and 172, respectively. The remaining mutations were nonsynonymous in the *VP2* region (Table 2).

From these results, it can be concluded that numerous mutations were found in both symptomatic and asymptomatic dogs at sites 172, 212, 227, and 236, with the exception of the amino acid alanine (A) > threonine (T) mutation at site 155. However, mutations at sites 247, 249, 260, 16, 31, and 34 were found only in symptomatic patients.

## DISCUSSION

This study revealed numerous genetic mutations in the polyprotein-encoding genes encoding CPV-2 capsid proteins 1 and 2. The latter gene is an important encoded gene for host and tissue tropisms and immunogenicity [11–13]. Demonstrating the role of certain genes in the pathogenicity of the virus could help scientists in designing suitable vaccines considering the genetic status of the virus in local isolates in Iraq.

Several researchers have reported that genetic variability in the *VP2* gene, which results in amino acid substitutions, likely affects the pathogenicity of CPV-2 [14, 15]. Our study detected numerous mutations by sequencing the *VP1* and partial *VP2* sequences and the intergenic sequence. Most of these mutations occur in *VP2*. However, few of these mutations lead to amino acid changes. This could explain the role of these mutations in triggering disease in domestic dogs compared with stray dogs [14, 16, 17]. The sequencing of *VP2* has been performed in numerous studies to demonstrate the genetic role of CPV-2 worldwide [18–22] and in Iraq [3, 7, 8, 19]. Temizkan and Sevinc Temizkan [6] reported that some CPV-2 strains have been transmitted from Iraq to Turkey. A phylogenetic study conducted in Turkey revealed three variants of the virus genetically associated with strains originating from China and Indonesia [10]. The disease is also highly distributed in Iran and has various genetic variants [23, 24]. Therefore, efficient immunological analysis must be performed before any vaccination schedule [25].

A previous study by Alam *et al*. [26] reported a high homology of isolates from Bangladesh to CPV-2 strains originating from Iraq. Along with VP1, this gene is responsible for antigenic shifting since the discovery of CPV-2, and four antigenic strains, namely CPV-2, CPV-2a, CPV-2b, and CPV-2c, have been identified [27, 28].

In the present study, the genetic variance detected in symptomatic domestic dogs compared with the genetic mutations within the asymptomatic dogs could explain how genetic polymorphisms affect the severity of infection, with the possibility of linking these mutations to the clinical severity; however, more samples are needed to determine the role of these mutated sites in phenotypic changes. In addition, analysis of other genes, such as non-structural genes, is needed to demonstrate the development of infection and disease pathogenicity. Most importantly, this study revealed that asymptomatic hosts can play an important role in the dissemination of CPV-2 into the environment.

## CONCLUSION

The study highlights significant genetic variations in the *VP1* and *VP2* genes of CPV-2 from symptomatic and asymptomatic cases in Iraq, emphasizing the role of mutations, particularly in *VP2*, in influencing disease virulence and clinical severity. Symptomatic cases exhibited more frequent and impactful mutations, with potential implications for vaccine effectiveness and virus control, while asymptomatic hosts were identified as reservoirs contributing to environmental dissemination. However, the study is limited by its sample size, geographical focus, and scope, as it did not explore other viral genes or phenotypic impacts of mutations nor did it include environmental or wild host samples. Future research should focus on whole-genome sequencing to capture greater genetic diversity, functional studies to assess phenotypic impacts of mutations, and the development of region-specific vaccines tailored to the genetic variations identified. Longitudinal studies and investigations into asymptomatic hosts’ roles in viral spread are crucial for comprehensive disease control and management strategies. This foundational work underscores the need for targeted approaches to effectively mitigate CPV-2 in endemic regions.

## AUTHORS’ CONTRIBUTIONS

MA: Sample collection and preparation, primer design and PCR, and drafted the manuscript. AHA: conducted the genetic analysis and phylogenetic analysis. AN: Data analysis and revised the manuscript. All authors have read and approved the final manuscript.

## References

[1] Ukwueze C.S, Akpan E.S, Ezeokonkwo R.C, Nwosuh C.I, Anene B.M (2020). Haematological, oxidative stress and electrolyte alterations in puppies with canine parvoviral enteritis. Iraqi J. Vet. Sci.

[2] Cotmore S.F, Agbandje-McKenna M, Canuti M, Chiorini J.A, Eis-Hubinger A.M, Hughes J, Mietzsch M, Modha S, Ogliastro M, Pénzes J.J, Pintel D.J, Qiu J, Soderlund-Venermo M, Tattersall P, Tijssen P, Ictv Report Consortium (2019). ICTV virus taxonomy profile:Parvoviridae. J. Gen. Virol.

[3] Zenad M.M, Radhy A.M (2020). Clinical, serological and antigenic study of feline panleukopenia virus in cats in Baghdad, Iraq. Iraqi J. Vet. Sci.

[4] Schirò G, Mira F, Canuti M, Vullo S, Purpari G, Chiaramonte G, Di Bella S, Cannella V, Randazzo V, Castronovo C, Vicari D, Guercio A (2022). Identification and molecular characterization of a divergent Asian-like canine parvovirus type 2b (CPV-2b) strain in Southern Italy. Int. J. Mol. Sci.

[5] Singh P, Kaur G, Chandra M, Dwivedi P.N (2021). Prevalence and molecular characterization of canine parvovirus. Vet. World.

[6] Temizkan M.C, Sevinc Temizkan S (2023). Canine parvovirus in Turkey:First whole-genome sequences, strain distribution, and prevalence. Viruses.

[7] Miranda C, Thompson G (2016). Canine parvovirus:The worldwide occurrence of antigenic variants. J. Gen. Virol.

[8] Abas Z.A, Omer Baba Sheikh M, Aziz H.N, Abid O.I (2022). Genetic diversity and genotyping of canine parvovirus type 2 by using the full-length *VP2* gene in North Iraq. Adv. Anim. Vet. Sci.

[9] Parrish C.R, Voorhees I.E.H, Hafenstein S.L (2021). Parvoviruses of carnivores, and the emergence of canine parvovirus (parvoviridae). In:Encyclopedia of Virology.

[10] Polat P.F, Şahan A, Aksoy G, Timurkan M.O, Dinçer E (2019). Molecular and restriction fragment length polymorphism analysis of canine parvovirus 2 (CPV-2) in dogs in Southeast Anatolia, Turkey. Onderstepoort J. Vet. Res.

[11] Karapinar Z, Karaman M, Kisadere İ, Usta M, Timurkan M.Ö (2023). Virological and pathological investigation of canine parvovirus-2 (CPV-2) with the assessment of the genetic variability of field strains. Turk. J. Vet. Anim. Sci.

[12] Li S, Chen X, Hao Y, Zhang G, Lyu Y, Wang J, Liu W, Qin T (2022). characterization of the VP2 and NS1 genes from canine parvovirus type 2 (CPV-2) and feline panleukopenia virus (FPV) in Northern China. Front. Vet. Sci.

[13] Alexis V.A, Sonia V, Daniela S, Miguel G, Timothy H, Valentina F, Lisette L, Leonardo S (2021). Molecular analysis of full-length VP2 of canine parvovirus reveals antigenic drift in CPV-2b and CPV-2c variants in central Chile. Animals (Basel).

[14] Voorhees I.E.H, Lee H, Allison A.B, Lopez-Astacio R, Goodman L.B, Oyesola O.O, Omobowale O, Fagbohun O, Dubovi E.J, Hafenstein S.L, Holmes E.C, Parrish C.R (2019). Limited intrahost diversity and background evolution accompany 40 years of canine parvovirus host adaptation and spread. J. Virol.

[15] Lina Z, Kai W, Fuyu A, Dongliang Z, Hailing Z, Xuelin X, Ce G, Hongmei Y, Yingjie K, Zhidong Z, Rongguang L, Yan H (2022). Fatal canine parvovirus type 2a and 2c infections in wild Chinese pangolins (*Manis pentadactyla*) in Southern China. Transbound. Emerg. Dis.

[16] Franzo G, De Villiers L, De Villiers M, Ravandi A, Gyani K, Van Zyl L, Coetzee L.M, Khaiseb S, Molini U (2022). Molecular epidemiology of Canine parvovirus in Namibia:Introduction pathways and local persistence. Prev. Vet. Med.

[17] Spera C.G, Lorenzetti E, Lavorente F.L.P, de Calasans Marques G, Bisca J.M, Teixeira C.R, Alfieri A.A, Alfieri A.F (2020). Canine parvovirus 2b in fecal samples of asymptomatic free-living South American coatis (*Nasua nasua*, Linnaeus, 1766). Braz. J. Microbiol.

[18] Silva L.M.N, Santos M.R, Carvalho J.A, Carvalho O.V, Favarato E.S, Fietto J.L.R, Bressan G.C, Silva-Júnior A (2022). Molecular analysis of the full-length VP2 gene of Brazilian strains of canine parvovirus 2 shows genetic and structural variability between wild and vaccine strains. Virus Res.

[19] Baba Sheikh M, Rashid P, Marouf A, Raheem Z, Manjunath S, Janga S (2017). Molecular typing of Canine parvovirus from Sulaimani, Iraq and phylogenetic analysis using partial VP2 gene. Bulg. J. Vet. Med.

[20] De la Torre D, Mafla E, Puga B, Erazo L, Astolfi-Ferreira C, Ferreira A.P (2018). Molecular characterization of canine parvovirus variants (CPV-2a, CPV-2b, and CPV-2c) based on the VP2 gene in affected domestic dogs in Ecuador. Vet. World.

[21] Magouz A, El-Kon I, Raya-Álvarez E, Khaled E, Alkhalefa N, Alhegaili A.S, El-Khadragy M.F, Agil A, Elmahallawy E.K (2023). Molecular typing of canine parvovirus type 2 by VP2 gene sequencing and restriction fragment length polymorphism in affected dogs from Egypt. Front. Microbiol.

[22] Zienius D, Lelešius R, Kavaliauskis H, Stankevičius A, Šalomskas A (2016). Phylogenetic characterization of canine parvovirus VP2 partial sequences from symptomatic dogs'samples. Pol. J. Vet. Sci.

[23] Morovvati A, Keyvanfar H, Zahraei Salehi T, Mousavi Nasab S.D, Zargar M (2022). Detection of Canine parvovirus type 2 by designing multiple methods and genetic characterization in Iran. Arch. Razi Inst.

[24] Ghajari M, Pourtaghi H, Lotfi M (2021). Phylogenetic analysis of canine parvovirus 2 subtypes from diarrheic dogs in Iran. Iran. J. Vet. Res.

[25] Shams F, Pourtaghi H, Abdolmaleki Z (2022). The first evaluation of the effectiveness of canine vaccination schedule by two commercial vaccines in Iran. BMC Vet. Res.

[26] Alam S, Chowdhury Q.M.M.K, Roy S, Chowdhury M.S.R, Hasan M, Mamun M.A, Uddin M.B, Hossain M.M, Rahman M.M, Rahman M.M (2021). Molecular detection and phylogenetic analysis of Canine Parvovirus (CPV) in diarrhoeic pet dogs in Bangladesh. Vet. Anim. Sci.

[27] De Oliveira Santana W, Silveira V.P, Wolf J.M, Kipper D, Echeverrigaray S, Canal C.W, Truyen U, Lunge V.R, Streck A.F (2022). Molecular phylogenetic assessment of the canine parvovirus 2 worldwide and analysis of the genetic diversity and temporal spreading in Brazil. Infect. Genet. Evol.

[28] Carrai M, Decaro N, Van Brussel K, Dall'Ara P, Desario C, Fracasso M, Šlapeta J, Colombo E, Bo S, Beatty J.A, Meers J, Barrs V.R (2021). Canine parvovirus is shed infrequently by cats without diarrhoea in multi-cat environments. Vet. Microbiol.

